# Efficacy of navigating through the intraplaque route using AnteOwl WR intravascular ultrasound in femoropopliteal chronic total occlusion

**DOI:** 10.1186/s42155-021-00228-4

**Published:** 2021-05-03

**Authors:** Naoki Hayakawa, Satoshi Kodera, Keisuke Takanashi, Shuichi Sahashi, Sandeep Shakya, Junji Kanda

**Affiliations:** 1 Department of Cardiovascular Medicine, Asahi General Hospital, I-1326 Asahi, Chiba, 289-2511 Japan; 2 Department of Cardiovascular Medicine, University of Tokyo Hospital, Tokyo, Japan

**Keywords:** Chronic total occlusion, Endovascular therapy, Intravascular ultrasound, AnteOwl WR

## Abstract

**Background:**

There is no consensus on the optimal guidewire passage route for femoropopliteal (FP) chronic total occlusion (CTO). If intraplaque wiring can be performed, a stent-less strategy using a drug-coated balloon can be realized even with FP CTO, and there is a high possibility that good expansion can be obtained even when stent deployment is performed. AnteOwl WR (AnteOwl) is a novel intravascular ultrasound (IVUS) device useful for navigating the second guidewire into the intraplaque route under IVUS observation from the subintimal space. Here, we describe representative cases of FP CTO in which CTO-specific IVUS was extremely useful.

**Case presentation:**

Case 1 involved a 79-year-old man with total occlusion of the left superficial femoral artery (SFA). We used a contralateral antegrade approach, but the guidewire was advanced into the subintimal space. We advanced AnteOwl into the CTO. By utilizing the asymmetric structure of the transducer and the IVUS wire, we were able to reflect the positional relationship among the IVUS transducer, IVUS wire, and target plaque onto the angiographic image. By aiming the wiring in that direction, we succeeded in traversing the center of the plaque and finally succeeded in obtaining good expansion using the drug-coated balloon. Case 2 involved a 76-year-old woman with total occlusion from the SFA to the popliteal artery. We used an ipsilateral antegrade approach. When AnteOwl was placed on the wire and advanced to the popliteal artery, the subintimal space in the middle of the SFA could be visualized. We employed an IVUS-guided parallel wiring technique and succeeded in passing through all intraplaque routes. Although the CTO was long, we could easily advance through the intraplaque route by reflecting the information obtained from AnteOwl in angiography.

**Conclusions:**

AnteOwl is an effective IVUS for FP CTO and facilitates a complex IVUS-guided procedure.

## Background

Developments in endovascular therapy (EVT) led to one of the first-line treatment strategies for femoropopliteal (FP) occlusive diseases (Norgen et al. 2007; Aboyans et al. 2017). Various techniques and devices have improved guidewire crossing and initial success for FP chronic total occlusion (CTO) (Kawasaki et al. 2008; Urasawa et al. 2014; Kitrou et al. 2015; Tan et al. 2017; Hayakawa et al. 2020). Some studies have reported the usefulness of performing an intraluminal approach with intravascular ultrasound (IVUS) guidance (Mori et al. 2017; Tsubakimoto et al. 2020). Alternatively, IVUS-guided intraplaque wiring requires high technical skills, and problems such as procedure time and the number of wires tend to be highlighted.

**Fig. 1:**
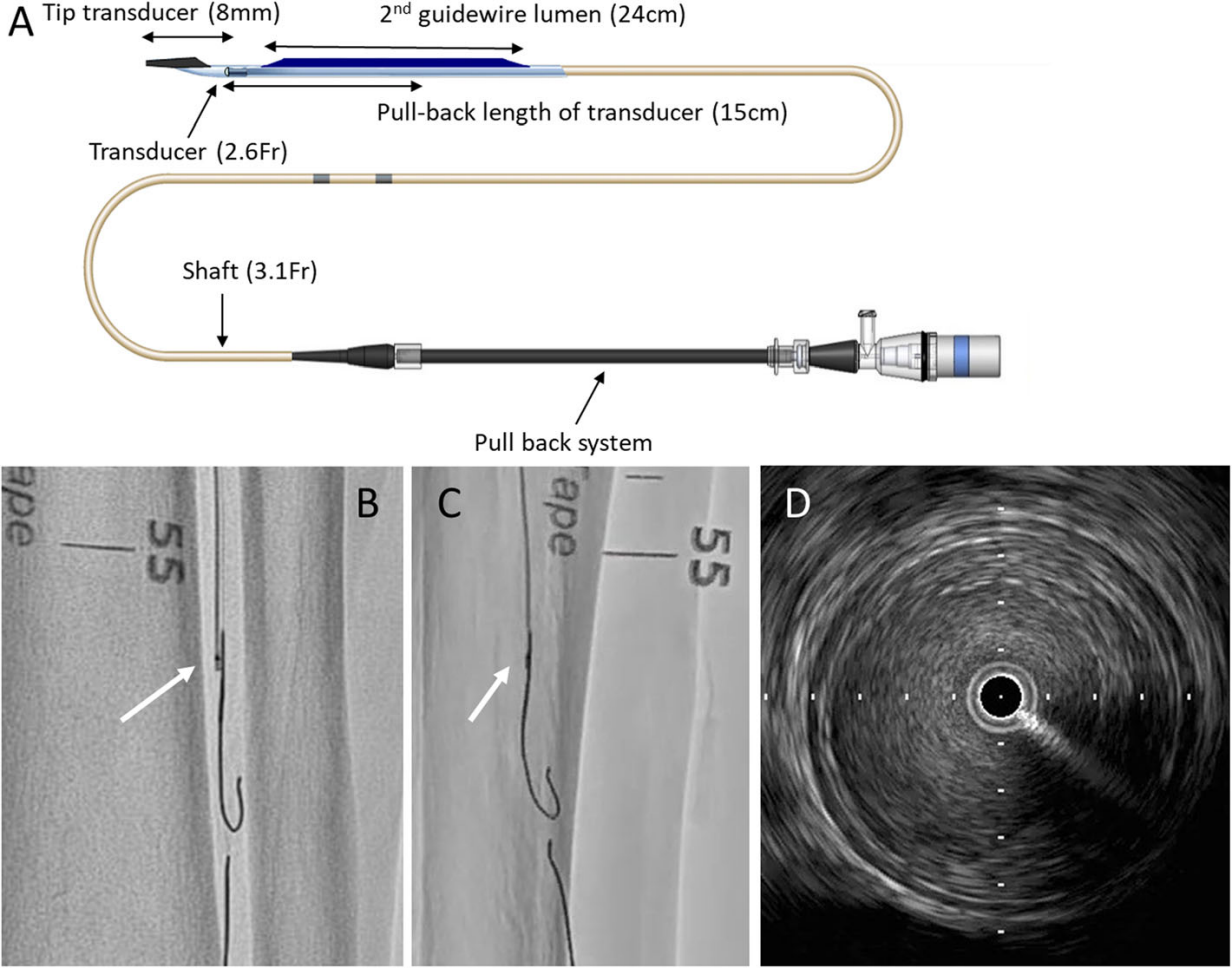
**a** Structure of AnteOwl WR (AnteOwl) intravascular ultrasound (IVUS). **b, c** Angiographic images of AnteOwl IVUS. **b** is the right anterior oblique view, and the transducer is to the left of the IVUS wire. Alternatively, **c** is the left anterior oblique view, and the transducer and IVUS wire are almost the same. The positional relationship between the transducer and IVUS wire can be understood in this way using rotational angiography. **d** Typical image of a superficial femoral artery lesion on AnteOwl IVUS

AnteOwl WR (AnteOwl) (TERUMO, Tokyo, Japan) is a novel IVUS device that is specialized in CTO (Fig. 1a). The transducer profile is small (2.6 Fr), and the length from the tip to the transducer is short (8 mm). Durable coating and the long proximal guidewire lumen improve crossability within the CTO. We can easily convert IVUS images into angiographic images for navigating the second guidewire through an intraplaque route under IVUS observation from the subintimal space using the asymmetrical structure of the transducer and IVUS guidewire (Fig. 1b, c). This IVUS device has a pullback transducer system, and the frequency is 40 MHz; this can be evaluated sufficiently in a peripheral artery (Fig. 1d). We have used Eagle-Eye Platinum ST IVUS (Philips, Amsterdam, The Netherlands) and Navifocus WR IVUS (TERUMO) mainly for EVT. Eagle-Eye Platinum IVUS has excellent pushability, but the tip has a symmetrical structure, and converting IVUS images into angiographic images was difficult. In addition, it can only produce 20-MHz images, which contains blood vessel information that is inferior to that shown in 40-MHz images. Navifocus WR IVUS is the predecessor of AnteOwl, and its structure is similar to that of AnteOwl. However, AnteOwl has a shorter distance from the tip to the transducer and has an improved coating, which makes it more convenient to use in navigating through CTOs. In addition, AnteOwl has a pullback transducer system, making wiring within CTOs much easier.

The usefulness of AnteOwl has been reported in the coronary CTO field (Okamura et al. 2020; Tanaka et al. 2020), but not in the EVT field. Therefore, here we describe representative cases of FP CTO in which AnteOwl was useful.

## Case presentations

### Case 1

**Fig. 2:**
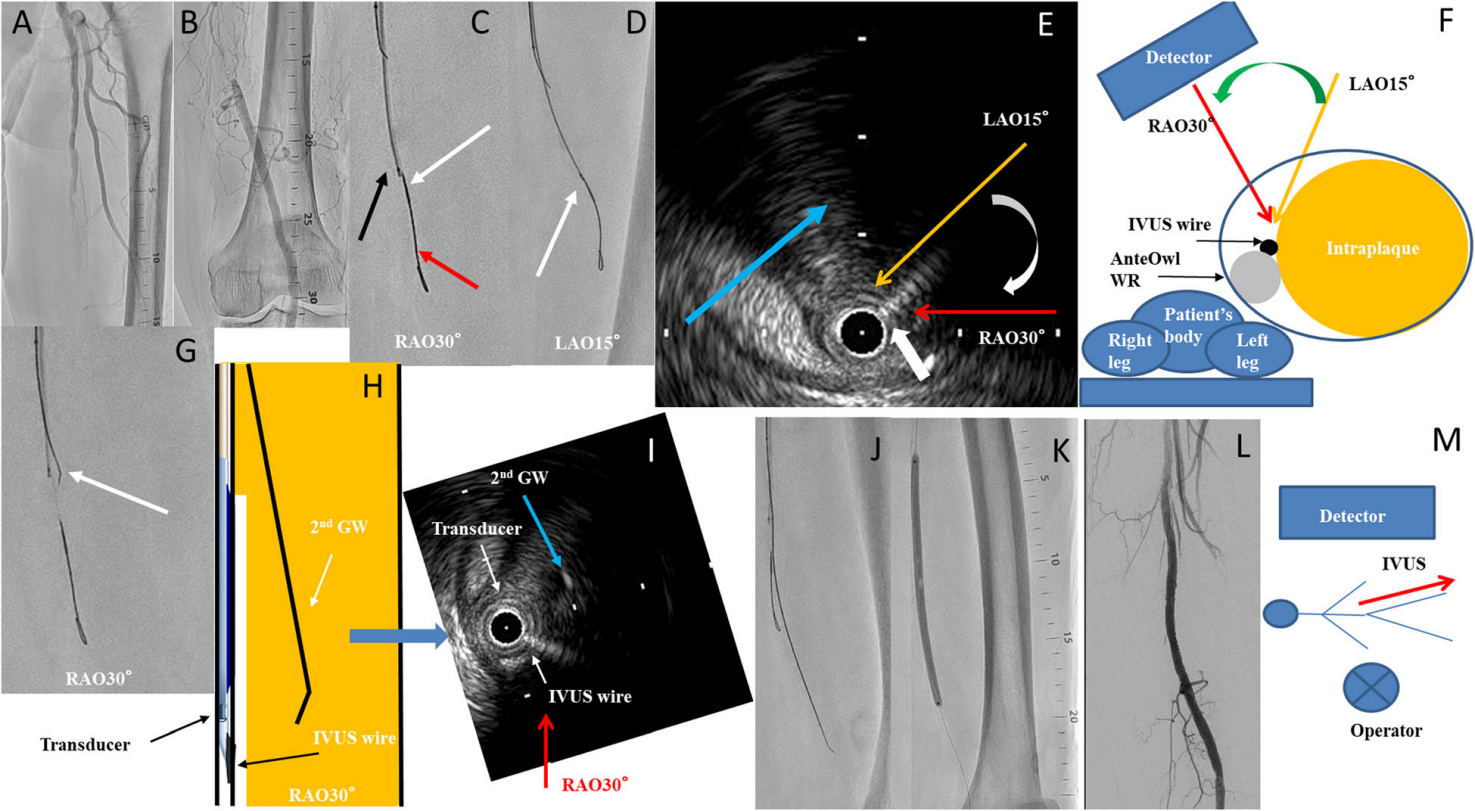
**a, b** Control angiography showed a tandem stenotic lesion in the left proximal superficial femoral artery and total occlusion of the left middle to distal aspects of the superficial femoral artery. **c** A 0.014-in. guidewire was advanced into the subintimal space (red arrow shows the tip of the guidewire). The black arrow shows the intravascular ultrasound (IVUS) transducer, and the white arrow shows the IVUS wire. The transducer was on the left side of the IVUS wire. **d** The transducer almost overlapped the IVUS wire (white arrow). On the basis of these findings, we confirmed that the IVUS transducer was at the bottom and the IVUS wire was at the top. **e** IVUS findings of the chronic total occlusion lesion. The white arrow shows the IVUS wire. The detector direction where the transducer and IVUS wire coincided on the angiographic image was left anterior oblique (LAO) 15° (yellow arrow). The detector direction where the transducer and target plaque were maximally separated at right anterior oblique (RAO) 30° (red arrow) was at 45° rotation to a clockwise direction from LAO 15° to RAO 30° (white arc arrow). The center of the target plaque was a few millimeters to the right of IVUS (blue arrow). **f** Cross-sectional image from the distal position (operator’s position). The IVUS wire and transducer coincided with LAO 15° and RAO 30° was maximally separated of IVUS and target plaque. The IVUS wire was on the surface side of the transducer, and the target plaque was on the right side of the IVUS catheter. **g** The second guidewire was advanced a few millimeters to the right of IVUS on the angiography (white arrow). **h**, **i** By rotating RAO 30° on the IVUS image to 6 o’clock according to the direction of guidewire advancement, the angiographic image and the IVUS image were visually matched. The IVUS catheter was in the subintimal space. The second guidewire was advanced a few millimeters to the right of the IVUS catheter on angiography, and the right blue arrow shows that the second guidewire could be advanced almost into the center of the target intraplaque space in the IVUS image. **j** The second guidewire could be passed through the chronic total occlusion lesion. **k, l** We dilated the drug-coated balloon. Final angiography showed a good outcome. **m** Positional relationship between patient and operator and direction of IVUS

A 79-year-old man undergoing hemodialysis owing to diabetes mellitus presented with claudication of the left lower limb. The patient’s ankle–brachial index was 0.70 on the left side. A 6-Fr guiding sheath (Destination® guiding sheath; TERUMO) was inserted into the right common femoral artery via the contralateral approach. Control angiography showed a tandem stenotic lesion in the left proximal superficial femoral artery (SFA) and total occlusion of the left middle to distal aspects of the SFA (Fig. 2a, b). First, a 0.014-in. guidewire (Gladius MGES® guidewire; Asahi Intec, Aichi, Japan) and 2.6-Fr microcatheter (Ichibanyari PAD2® microcatheter; Kaneka, Tokyo, Japan) seemed to be advanced into the subintimal space. We advanced AnteOwl (AnteOwl WR® IVUS; TERUMO) into the CTO. IVUS showed that the guidewire was in the subintimal space proximal to the CTO. We decided to perform the IVUS-guided parallel wiring technique using AnteOwl. We converted the direction of IVUS findings to angiography using the following five steps. (1) First, we performed rotational angiography from the right anterior oblique (RAO) 40° view to left anterior oblique (LAO) 40° view, the direction of which was the orthogonal axis against the CTO, to identify the upper side of the transducer and IVUS wire. Because the AnteOwl has an asymmetric transducer and IVUS wire, we were able to determine which was above and which was below from rotational angiography at an angle perpendicular to the IVUS catheter to see which was above. (2) The transducer was on the left side in the RAO 30° view, and the transducer and IVUS wire almost overlapped in the LAO 15° view (Fig. 2c, d). Based on these findings, we confirmed that the transducer was at the bottom and the IVUS wire was at the top. (3) The detector direction at which the transducer and IVUS wire coincided on the angiographic image was LAO 15° (Fig. 2e). (4) Next, the detector direction at which the transducer and target plaque were maximally separated was RAO 30°, which was at 45° rotation to the clockwise direction from LAO 15° to RAO 30° (Fig. 2e). (5) The IVUS findings revealed a target true lumen at which to aim on the right side of the IVUS catheter at RAO 30° (Fig. 2e, f). By rotating RAO 30° on the IVUS image to 6 o’clock according to the direction of guidewire advancement, the angiographic image and the IVUS image were visually matched (Fig. 2h, i). We advanced a 0.014-in. guidewire (Astato XS9–40® guidewire; Asahi Intec) and 2.6-Fr microcatheter (Ichibanyari PAD2® microcatheter; Kaneka) a few millimeters to the right (LAO side) of IVUS on angiography, and we advanced through the intraplaque route and succeeded in penetrating the lesion (Fig. 2g–i). After the predilation, we dilated a 6.0- × 150-mm drug-coated balloon (In.Pact Admiral®; Medtronic, Minneapolis, MN, USA) at the SFA lesion to prevent restenosis (Fig. 2i, j). The final angiography showed good antegrade flow with no dissection or residual stenosis (Fig. 2k).

### Case 2

**Fig. 3:**
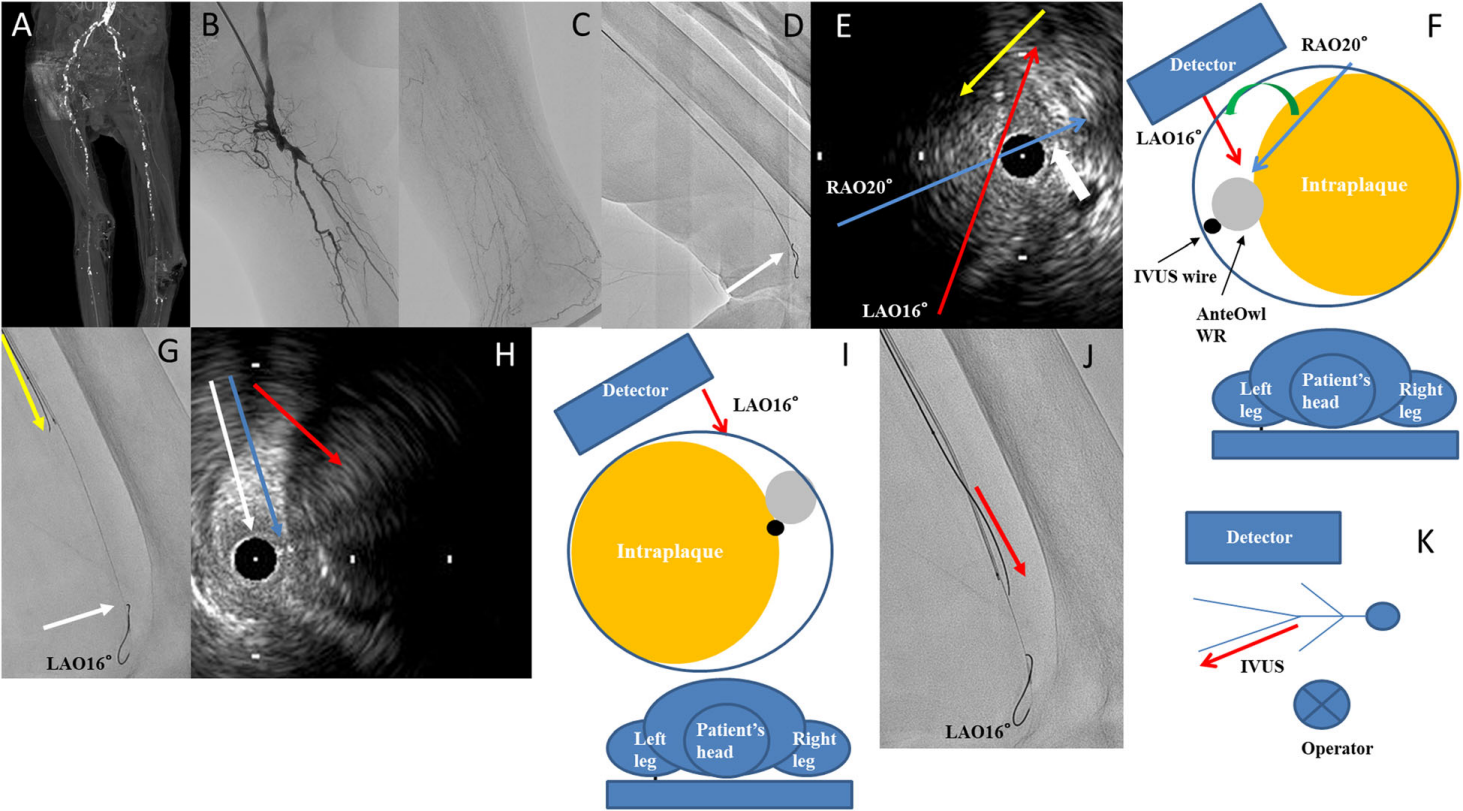
**a** Preprocedural enhanced computed tomography. **b, c** Control angiography showed total occlusion from the proximal aspect of the superficial femoral artery to the popliteal artery. The distal true lumen of the popliteal artery was minute (white arrow). **d** AnteOwl WR intravascular ultrasound (AnteOwl IVUS) could be advanced to the popliteal artery (white arrow). **e** IVUS findings of a superficial femoral artery distal lesion. The IVUS catheter was in the subintimal space. The white arrow shows the IVUS wire. The IVUS wire and transducer coincided with right anterior oblique 20° (blue arrow), and left anterior oblique 16° was maximally separated of IVUS and target plaque (red arrow). The yellow arrow shows that the direction of the second guidewire should be advanced. **f** Cross-sectional image from the proximal position (operator position). The transducer was on the surface side and the target plaque was on the right side of the IVUS catheter. **g** We converted the intravascular ultrasound (IVUS) findings into an angiographic image. The IVUS wire was on the right side (left anterior oblique side), the transducer was in the center, and the second guidewire was on the left side (right anterior oblique side) on angiography; these findings are almost the same as the IVUS findings in **e** White arrow shows the tip of AnteOwl WR IVUS. **h, i** IVUS findings of the popliteal artery lesion. The blue arrow shows the IVUS wire. The yellow arrow shows the IVUS transducer. The red arrow shows the direction of the target plaque at which we aimed. The target plaque was on the left side of the IVUS catheter. **j** The IVUS wire was in the center, the transducer was on the left side, the second guidewire was on the right side (left anterior oblique side) on angiography; these findings are almost the same as the IVUS findings. **k** Positional relationship between patient and operator and direction of IVUS

**Fig. 4:**
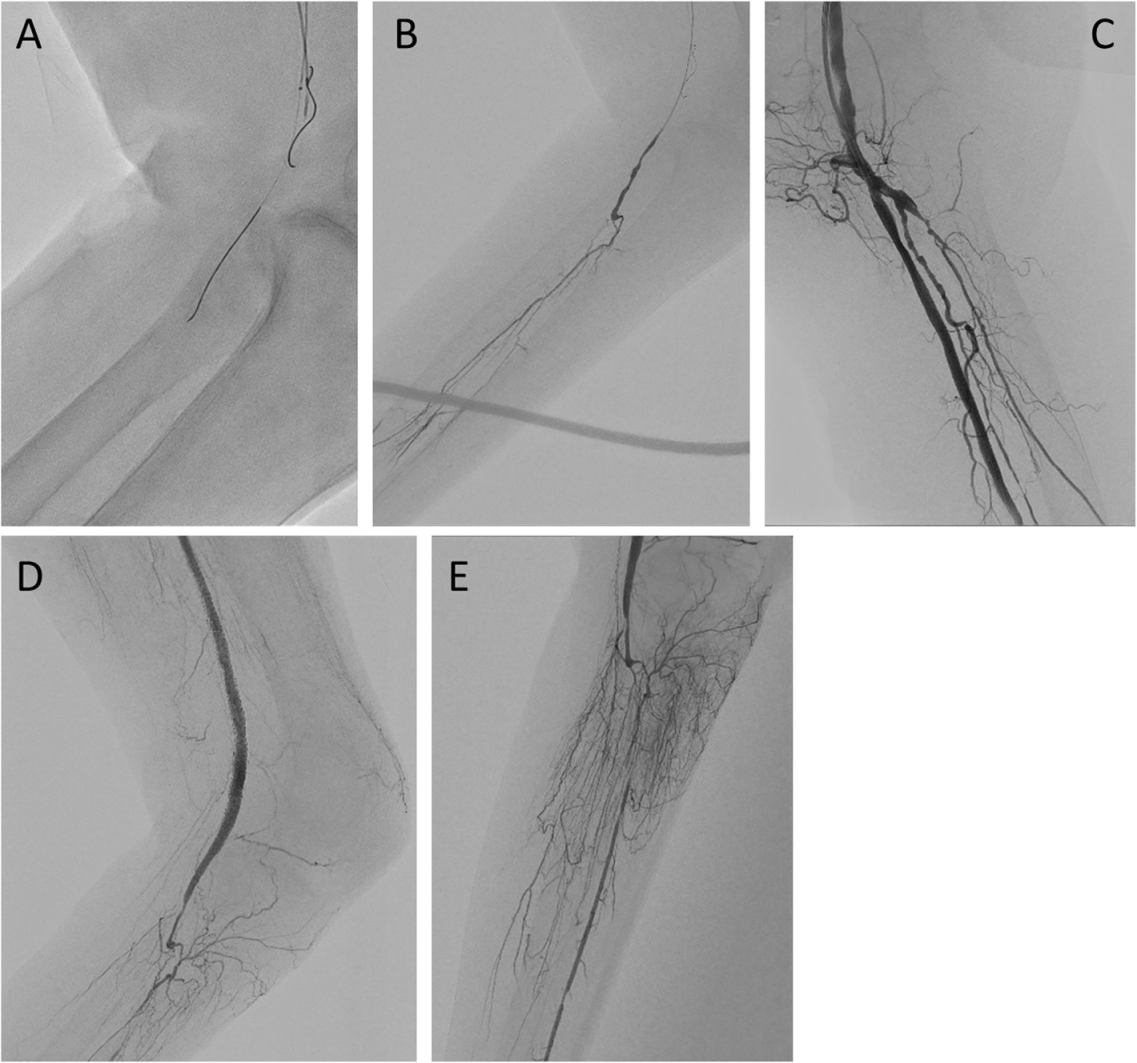
**a**, **b** A guidewire could be passed to the chronic total occlusion lesion. We confirmed this using tip injection with a micro catheter. **c–e** Final angiography shows that the lumen from the superficial femoral artery to the popliteal artery was well opened

A 76-year-old woman with hypertension and old cerebral hemorrhage presented with ulceration and gangrene of the left foot. Preoperative contrast-enhanced computed tomography showed total occlusion beyond the left external iliac artery (Fig. 3a). First, we treated the external iliac artery to the common femoral artery using a transradial approach. Next, we treated FP CTO using an ipsilateral antegrade approach. When performing EVT on the ipsilateral side, the posture was reversed from the standard position, with the left-hand side of the operator on the foot side and the right-hand side on the head side (Fig. 3k). Control angiography showed total occlusion from the SFA proximal to the popliteal artery (Pop A) (Fig. 3b, c). We advanced a 0.014-in. guidewire (Gladius MGES® guidewire; Asahi Intec) and 2.6-Fr microcatheter (Ichibanyari PAD2® microcatheter; Kaneka) to the Pop A. We advanced AnteOwl IVUS into the CTO (Fig. 3d). Because it was a subintimal route from the distal aspect of the SFA shown in the IVUS findings, we performed an IVUS-guided parallel wiring technique through the intraplaque route. AnteOwl had a pullback system; thus, performing the parallel wiring technique in real time was possible while observing the lesion in front without inserting and removing the IVUS catheter advanced to the distal aspect of the CTO. By reflecting the IVUS findings into an angiographic image in the same manner as in Case 1, we performed the following steps. From rotational angiography, the transducer was on the upper side. The IVUS-wire and transducer coincided with about RAO 20°. And LAO 16° was maximally separated of IVUS and target plaque. We found that the left side (RAO side) of the IVUS catheter on angiography should be aimed at the distal aspect of the SFA (Fig. 3e–g). IVUS showed the 1st guidewire had advanced spirally in the proximal part of the Pop A, and we found that the right side (LAO side) of the IVUS catheter (opposite the distal SFA) should be aimed at the Pop A (Fig. 3h–j). We advanced a 0.014-in. guidewire (Astato XS9–40® guidewire; Asahi Intec) and 2.6-Fr microcatheter (Ichibanyari PAD2® microcatheter; Kaneka) and succeeded in accessing the lesion (Fig. 4 a, b). After the predilation, we deployed three drug-eluting stents (Eluvia® drug-eluting stent; Boston Scientific, Marlborough, MA, USA) from the SFA to the Pop A. The final angiography showed good antegrade flow (Fig. 4 c–e).

## Discussion

We demonstrated the feasibility of EVT for FP CTO using the AnteOwl IVUS-guided approach. In these cases, we could pass through all intraplaque routes via an antegrade approach despite the complexity of the CTO lesion. Studies have reported the use of AnteOwl IVUS in the coronary artery CTO field (Okamura et al. 2020; Tanaka et al. 2020); however, to the best of our knowledge, this is the first case report on the use of AnteOwl IVUS in the EVT field. In the coronary artery CTO field, a method for advancing a guidewire by IVUS has been reported (Matsubara et al. 2004; Okamura et al. 2010; Okamura et al. 2014). However, in the EVT field, no studies have explained specifically how to navigate guidewires through the intraplaque route using IVUS guidance.

In AnteOwl, the structures of the proximal transducer and IVUS wire are asymmetrical, facilitating conversion of IVUS images into angiographic images to allow accurate navigation of the second guidewire into the intraplaque route. Once the IVUS is advanced to the distal aspect of the CTO, the lesion can be observed without moving the IVUS itself in and out using the pullback transducer system. The pullback system eliminates the need to insert and remove the IVUS, prevents the expansion of a large subintimal space, causes less interference with the second guidewire, and is expected to shorten the procedure time. In Case 2, although the first guidewire progressed spirally in the subintimal space, accurately reflecting the IVUS findings into an angiographic image was easy; thus, despite the long CTO exceeding 30 cm, we succeeded in accessing the CTO using only the antegrade approach.

There is no consensus on the optimal guidewire passage route for FP CTO lesions (Soga et al. 2013; Mori et al. 2017). However, some clinical studies have recently reported that IVUS-guided wiring improves the clinical outcomes of EVT for FP CTO (Mori et al. 2017; Tsubakimoto et al. 2020). The advantages of intraplaque guidewire crossing are as follows: a stent-less strategy can be realized using a drug-coated balloon even for CTO lesions, it can be expanded reliably and safely even when a stent is implanted, and the antegrade guidewire passage may reduce the need for an extra distal puncture for the retrograde approach.

In our cases, the initial success of the procedure and good short-term prognosis were confirmed; however, the usefulness of AnteOwl IVUS-guided EVT in various cases and its long-term results remain unclear. A much larger study is required to confirm the efficacy of AnteOwl IVUS-guided EVT for FP CTO.

## Conclusions

AnteOwl IVUS was effective for FP CTO and enabled us to simplify complex IVUS-guided procedures. Using AnteOwl IVUS provides access to the complex CTO lesion via intraplaque routes.

## Data Availability

The datasets used and/or analyzed during the current study are available from the corresponding author on reasonable request.

## Abbreviations

FP: Femoropopliteal
CTO: Chronic total occlusion
IVUS: Intravascular ultrasound
SFA: Superficial femoral artery
Pop A: Popliteal artery
EVT: Endovascular therapy
RAO: Right anterior oblique
LAO: Left anterior oblique
